# Food Insecurity Associated With Higher Odds of Hypertriglyceridemic Waist Phenotype in Women: A Cross‐Sectional Study

**DOI:** 10.1002/fsn3.71244

**Published:** 2025-11-24

**Authors:** Paria Jadidi, Atieh Mirzababaei, Faezeh Abaj, Azadeh Dehghani, Neda Valisoltani, Moloud Ghorbani, Cain C. T. Clark, Khadijeh Mirzaei

**Affiliations:** ^1^ Department of Nutrition, SR.C Islamic Azad University Tehran Iran; ^2^ Chronic Diseases Research Center, Endocrinology and Metabolism Population Sciences Institute Tehran University of Medical Sciences Tehran Iran; ^3^ Department of Community Nutrition, School of Nutritional Sciences and Dietetics Tehran University of Medical Sciences Tehran Iran; ^4^ Department of Nutrition, Dietetics & Food Monash University Notting Hill Victoria Australia; ^5^ Micronutrient Research Center, Research Institute for Endocrine Disorders, Research Institute for Endocrine Sciences Shahid Beheshti University of Medical Sciences Tehran Iran; ^6^ Department of Nutrition, Faculty of Medicine Mashhad University of Medical Sciences Mashhad Iran; ^7^ Centre for Intelligent Healthcare Coventry University Coventry UK

**Keywords:** food insecurity, hypertriglyceridemic waist (HTGW), obesity

## Abstract

Food insecurity has been linked to obesity, central adiposity, and dyslipidemia, with women particularly vulnerable. These consequences align with the hypertriglyceridemic waist (HTGW) phenotype, a determinant of visceral adiposity and cardiometabolic risk. Although prior studies suggest associations between FI, waist circumference, and triglyceride levels, direct evidence on FI and HTGW remains scarce, especially in Middle Eastern populations. The present investigation assessed the potential link between food insecurity and the hypertriglyceridemic waist phenotype among overweight and obese Iranian women. In this cross‐sectional study, 250 overweight and obese women dietary intake was assessed using a validated 147‐item (FFQ), and household food security was evaluated with the 18‐item USDA (HFSS). Anthropometric measures and body composition were obtained via bioelectrical impedance analysis. Physical activity was assessed using the validated IPAQ. Participants were grouped based on waist circumference and triglycerides: WC < 88 cm and TG < 150 mg/dL were classified as normal waist and triglycerides, while WC ≥ 88 cm and TG ≥ 150 mg/dL were considered to have the hypertriglyceridemic waist (HTGW) phenotype. Analyses were performed in SPSS version 25. A significant positive association was found between food insecurity and the HTGW phenotype. In unadjusted analysis, food‐insecure women had higher odds of HTGW compared to food‐secure women (OR = 2.24, 95% CI =1.19–4.20, *p* = 0.01). After adjustment for age, BMI, total energy intake, and physical activity, the association remained significant (OR = 2.13, 95% CI = 1.06–4.28, *p* = 0.03). The findings demonstrate that food insecurity is significantly associated with the HTGW phenotype among overweight and obese women. As the first study to specifically examine this relationship in this population, these findings underscore food insecurity as a critical social determinant of metabolic risk, highlighting the need for future longitudinal studies and targeted interventions.

## Graphical abstract

In this cross‐sectional study of 250 overweight and obese Iranian women, food insecurity was significantly associated with higher odds of the hypertriglyceridemic waist (HTGW) phenotype. Women experiencing food insecurity had more than twice the likelihood of presenting the HTGW phenotype compared with food‐secure counterparts (adjusted OR = 2.13, 95% CI = 1.06–4.28). These findings highlight food insecurity as a social determinant of metabolic disruption and underscore the need for targeted nutritional and policy interventions. Graphical abstract illustrating behavioral and physiological pathways linking food insecurity to HTGW; adjusted (OR = 2.13 95% CI = 1.06–4.28). Adjusted for age, BMI, energy intake, and physical activity.

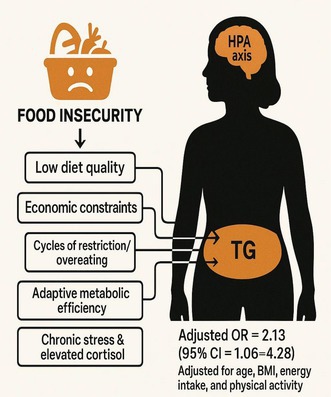

**Abbreviations**
ANCOVA: analysis of covariance
ANOVA: analysis of variance
BF%: body fat percentage
BFM: body fat mass
BIA: bioelectrical impedance analysis
BMI: body mass index
BP: blood pressure
CRP: C‐reactive protein
FBS: fasting blood sugar
FFM: fat‐free mass
FFQ: FFQ
HDL: high‐density lipoprotein cholesterol
HOMA: homeostatic model assessment
HTGW: hyper‐triglyceridemic waist
IL‐6: interleukin 6
IPAQ: international physical activity questionnaires
LDL: low‐density lipoprotein cholesterol
MET: metabolic equivalent
MetS: metabolic syndrome
SD: standard deviation
TC: total cholesterol
TG: triglyceride
USDA: United States Department of Agriculture
WC: waist circumference
WHR: waist hip ratio

## 1 Introduction

Inadequate access to food combined with unbalanced dietary habits represents a global health challenge, leaving billions of people at risk of malnutrition (Abbade and Dewes 2015). Food insecurity (FI) is defined as the uncertainty or restriction in obtaining adequate, safe, and nutritious food necessary to maintain health (IFAD UN 2017). The severity of FI can vary, from declines in food quality and quantity to recurrent hunger and skipped meals, and is largely influenced by socioeconomic and demographic factors such as unemployment, age, ethnicity, and limited education (Tabibian et al. 2018; Dave et al. 2009). Current global estimates indicate that more than one billion people fail to meet minimum energy and macronutrient needs, while nearly two billion individuals experience deficiencies in essential micronutrients (Barrett 2010). Women remain especially vulnerable to FI due to gender‐related disparities and the increasing number of households headed by women (Ivers and Cullen 2011). Data from Iran further underscore the seriousness of this issue, with a systematic review reporting FI prevalence rates of 49% among adolescents and 61% among mothers, levels notably higher than those observed in many other regions (Behzadifar et al. 2016). The impact of FI extends far beyond individual nutrition, playing a role in the development of long‐term health conditions and societal burdens. Persistent FI contributes to cardiovascular disease, type 2 diabetes, depression, and poor quality of life, underscoring its multidimensional impact on public health and the healthcare system (Zierath et al. 2023). Moreover, the economic costs of FI extend beyond individuals, placing a substantial strain on healthcare expenditures and social welfare programs (Chang et al. 2022).

In addition to its established role in undernutrition, FI can lead to increases in body weight, an effect frequently labeled the “food insecurity–obesity paradox” (Parker et al. 2010; Rasmusson et al. 2019). This pattern is most consistently observed among women, who appear more vulnerable to weight increases when experiencing food insecurity (Crespo‐Bellido et al. 2021). The earliest report of such a connection was provided by Dietz (1995), suggesting that recurring cycles of food scarcity may trigger both behavioral and physiological responses, including greater fat storage and poorer food choices (El Zein et al. 2020). These processes together offer a plausible explanation for the heightened prevalence of obesity in food‐insecure groups.

When access to affordable, nutrient‐dense foods like fruits and vegetables is limited, individuals often turn to cheaper, calorie‐dense options such as fast foods and sugar‐sweetened beverages. This dietary shift creates metabolic conditions that contribute to central obesity and elevated TG levels (Parikh and Mohan 2012). In contrast to conventional indicators such as BMI, which are unable to differentiate between fat mass and lean mass, the HTGW phenotype offers a more accurate reflection of visceral adiposity (Janghorbani et al. 2017; Zheng et al. 2022). Because of its simplicity, affordability, and strong predictive ability, HTGW is especially practical for both epidemiological research and clinical practice, particularly in low‐ and middle‐income settings where advanced diagnostic methods may not be available (Zheng et al. 2022).

The physiological changes described above significantly contribute to the onset of metabolic syndrome (MetS), which involves a cluster of metabolic abnormalities, including elevated blood pressure, impaired fasting glucose, high triglycerides, lowered HDL, and expansion of waist circumference (Wang et al. 2020). Within this context, the hypertriglyceridemic waist (HTGW) phenotype, defined by the simultaneous occurrence of enlarged waist circumference and raised triglyceride levels, serves as a validated marker of visceral fat accumulation and an enhanced cardiometabolic risk profile (Fernández‐García et al. 2020). Because FI, obesity, and lifestyle behaviors are modifiable risk factors, their role in shaping the HTGW phenotype warrants particular attention. Importantly, recent evidence highlights HTGW as a reliable predictor of cardiovascular disease, further underscoring its clinical relevance (Zheng et al. 2022). Evidence from Iran further illustrates the complex role of FI in influencing obesity‐related outcomes. In a cross‐sectional study of Tehran households, Mohammadi et al. (2013) indicated that severe FI contributed to a higher prevalence of central obesity, whereas moderate FI showed an inverse relationship with overweight in women. Similar associations have been reported elsewhere: women living in households with very low food security displayed significantly higher TG levels (Mohajeri and Mohajery 2022), while an analysis of U.S. women indicated a greater prevalence of waist circumference above 88 cm in low food security settings (Park and Strauss 2020). Comparable findings have also been documented in Asian populations, reinforcing the potential contribution of FI to adverse metabolic profiles (Mohajeri and Mohajery 2022).

Despite these observations, direct evidence linking FI to the HTGW phenotype in overweight and obese women remains limited, particularly within Middle Eastern populations. While several systematic reviews have documented associations between FI and cardiometabolic risk, to date, no research has explicitly examined the association between FI and HTGW among overweight Iranian women (Salinas‐Roca et al. 2022; Te Vazquez et al. 2021). Addressing this gap, the present study was designed to investigate the association between FI and the HTGW phenotype in a cohort of overweight and obese women.

## 2 Materials and Methods

### 2.1 Participants

This cross‐sectional investigation involved 250 women who were overweight or obese, enrolled from health centers in Tehran, Iran, between 2016 and 2017. Participants were females aged between 18 and 48 years, who possessed a BMI within the twenty‐five to forty kg/m^2^ range, who provided voluntary consent, and had no history of chronic conditions such as cancer, liver or kidney dysfunction, uncontrolled thyroid disorders, hypertension, or impaired glucose metabolism. Exclusion criteria encompassed the use of vitamin or mineral supplements, hormonal treatments, herbal medications, or steroids, as well as extreme energy intakes (< 800 kcal/day or > 4200 kcal/day). The study protocol received approval from the Ethics Committee of Tehran University of Medical Sciences (IR.TUMS.MEDICINE.REC.1402.172), ensuring that all participants gave written informed consent before being enrolled.

### 2.2 Anthropometric and Body Composition

Body weight was recorded to the nearest 0.1 kg using a digital calibrated scale (Seca, Hamburg, Germany), and height was measured using a wall‐mounted stadiometer (Seca, Hamburg, Germany) with a precision of 0.5 cm. Body mass index (kg/m^2^) was then calculated from these measurements. WC was determined at the midpoint between the lower rib margin and the iliac crest by a trained professional. Body composition parameters, including SLM, FFM, BFM, SMM, and BMR, were assessed using a multi‐frequency bioelectrical impedance analyzer (InBody 770, Seoul, South Korea). Fasting, absence of recent exercise, and removal of metallic items were required prior to the test.

### 2.3 Dietary Intake, Physical Activity, and Food Security

Participants' year‐long food consumption was evaluated by trained nutritionists employing a validated 147‐item FFQ. Nutrient intakes were calculated in grams using Nutritionist IV software. Physical activity was measured with the IPAQ, expressed in MET‐h/week and categorized as low (< 600), moderate (600–3500), or high (> 3500) (Craig et al. 2003; Moghaddam et al. 2012). Over the past year, trained staff collected information on households' access to sufficient food using the 18‐item USDA HFSS (Mohammadi et al. 2012), which had been tailored and validated for the Iranian population. It categorized households as secure or insecure (Savari et al. 2021). Scoring followed Gray and Bickel's methodology (Bickel et al. 2000), with total scores classifying participants into food‐secure or food‐insecure groups.

### 2.4 Biochemical and Clinical Measurements

A 10–12 h overnight fast preceded blood collection; samples were then centrifuged to separate the serum, which was maintained at −80°C for subsequent evaluation. Serum TG, TC, LDL, HDL, and FBS were assessed via enzymatic colorimetric kits (Pars Azmoon, Tehran, Iran). Serum insulin was determined via radioimmunoassay (DRG Pharmaceuticals, Germany), and high‐sensitivity C‐reactive protein (hs‐CRP) was quantified using an immunoturbidimetric assay. Aspartate aminotransferase (AST) and alanine aminotransferase (ALT) levels were measured following IFCC protocols. Insulin resistance was estimated using the HOMA‐IR formula: [Fasting plasma glucose (mmol/L) × Fasting plasma insulin (mIU/L)]/22.5 (Heshmati et al. 2020). Blood pressure and pulse were measured twice by a trained physician using a standardized sphygmomanometer (Omron, Germany) after a 15‐min rest. Hypertension was defined as systolic blood pressure ≥ 130 mmHg or diastolic blood pressure ≥ 85 mmHg (Bergler‐Klein 2019). Demographic data, including age, marital status, occupation, and educational level, were collected via structured interviews.

### 2.5 HTGW Phenotype

Participants were grouped based on waist circumference and triglyceride levels. Those with WC < 88 cm and TG < 150 mg/dL were classified as having a normal waist, normal triglyceride (NWNT). In contrast, those with WC ≥ 88 cm and TG ≥ 150 mg/dL were identified as having the HTGW phenotype (Amini et al. 2011).

### 2.6 Statistical Analysis

The Kolmogorov–Smirnov test assessed the normality of continuous variables. Between‐group differences were analyzed with independent *t*‐tests for numerical data and Chi‐square tests for categorical data. Adjusted comparisons of dietary intake were performed using ANCOVA, controlling for total caloric intake. Logistic regression analysis was employed to estimate the odds of having the HTGW phenotype in relation to food insecurity, controlling for age, BMI, energy intake, and physical activity. Analyses were performed in SPSS version 25, with *p* < 0.05 considered significant.

## 3 Results

### 3.1 Study Population Characteristics

This cross‐sectional study enrolled 250 women with overweight or obesity, with an average age of 36.67 ± 9.19 years. Participants had a mean body weight of 80.28 ± 11.05 kg and a body mass index (BMI) of 30.98 ± 3.90 kg/m^2^, with an average waist‐to‐hip ratio of 1.16 ± 4.54. Most participants (70.8%) were married, 46.8% held a bachelor's degree or higher, 39.9% reported favorable economic conditions, and 27.2% were employed. Based on phenotype classification, 91 women (36.4%) were identified with the HTGW phenotype, and 28.2% experienced food insecurity.

### 3.2 HTGW Phenotype and Participant Profile

As detailed in Table 1, unadjusted analyses revealed that the HTGW group had notably elevated levels for multiple parameters relative to the reference group, including body weight, BMI, waist circumference (WC), basal metabolic rate (BMR), fat‐free mass (FFM), skeletal muscle mass (SMM), soft lean mass (SLM), fasting blood sugar (FBS), insulin, HOMA‐IR, triglycerides (TG), total cholesterol (TC), low‐density lipoprotein (LDL), systolic blood pressure (SBP), alanine aminotransferase (ALT), and high‐sensitivity C‐reactive protein (hs‐CRP) (all *p* < 0.05). Conversely, the reference group was characterized by a significantly elevated average level of HDL (*p* = 0.02).

**TABLE 1:** Characteristics of the study population among the HTGW phenotype and normal groups (*N* = 250).

Variables	Total	Normal *N* = 159	HTGW *N* = 91	*p*	*p* *
Demographic variables					
Age (years)	36.67 ± 9.19	36.46 ± 9.01	38.16 ± 9.67	0.25	0.28
Anthropometric parameters					
Weight (kg)	80.28 ± 11.05	79.74 ± 11.11	84.27 ± 9.77	**0.005**	**0.001**
Height (cm)	161.22 ± 5.87	161.04 ± 5.85	162.55 ± 5.90	0.100	0.170
BMI (kg/m^2^)	30.98 ± 3.90	30.84 ± 3.93	32.02 ± 3.52	**0.030**	**0.010**
WHR (cm)	1.16 ± 4.54	1.19 ± 4.85	0.95 ± 0.04	0.35	**0.005**
WC (cm)	99.16 ± 9.42	98.57 ± 9.41	103.40 ± 8.41	**< 0.001**	**0.001**
Blood pressure					
SBP (mmHg)	111.73 ± 13.62	110.81 ± 13.56	116.33 ± 13.10	**0.01**	0.11
DBP (mmHg)	77.60 ± 10.40	76.73 ± 10.54	81.97 ± 8.47	**< 0.001**	**0.02**
Body composition					
BFM (kg)	34.74 ± 8.75	34.50 ± 8.84	36.40 ± 7.93	0.12	**0.04**
FFM (kg)	46.52 ± 5.71	46.13 ± 5.64	49.33 ± 5.50	**< 0.001**	**0.001**
SMM (kg)	25.56 ± 3.44	25.32 ± 3.40	27.23 ± 3.27	**< 0.001**	**< 0.001**
SLM (kg)	43.77 ± 5.44	43.39 ± 5.37	46.53 ± 5.20	**< 0.001**	**< 0.001**
BMR	1377.56 ± 134.66	1369.48 ± 134.88	1435.57 ± 118.98	**< 0.001**	**< 0.001**
Biochemical biomarkers					
FBS (mg/dL)	87.49 ± 9.64	86.68 ± 8.71	90.91 ± 12.41	**0.02**	**0.02**
TC (mg/dL)	185.30 ± 35.77	180.08 ± 34.63	207.32 ± 32.22	**< 0.001**	**< 0.001**
TG (mg/dL)	118.10 ± 58.88	96.11 ± 35.37	209.63 ± 48.01	**< 0.001**	**< 0.001**
HDL (mg/dL)	46.58 ± 10.86	47.35 ± 10.75	43.32 ± 10.81	**0.02**	**0.009**
LDL (mg/dL)	95.30 ± 24.12	91.88 ± 23.20	109.73 ± 22.76	**< 0.001**	**< 0.001**
Insulin (mlU/mL)	1.21 ± 0.23	1.19 ± 0.22	1.30 ± 0.24	**0.008**	**0.009**
AST (mg/dL)	18.05 ± 7.75	17.55 ± 7.26	20.18 ± 9.33	0.07	**0.001**
ALT (mg/dL)	19.49 ± 13.83	18.41 ± 12.82	24.08 ± 16.88	**0.03**	**0.002**
Inflammatory biomarkers					
hs‐CRP (mg/dL)	4.34 ± 4.62	3.86 ± 4.29	6.43 ± 5.40	**0.004**	**0.005**
HOMA Index	3.35 ± 1.27	3.17 ± 1.12	4.14 ± 1.56	**< 0.001**	**0.05**
Marital status					
Single	73 (29.2)	58 (36.5)	15 (16.5)	0.14	0.16
Married	177 (70.8)	101 (63.5)	76 (83.5)		
Education					
Illiterate	33 (13.1)	12 (7.5)	21 (23.1)	0.28	0.25
Diploma	95 (37.9)	59 (37.1)	36 (39.6)		
Bachelor	122 (46.8)	88 (55.3)	34 (37.4)		
Job					
Unemployed	143 (57.2)	92 (57.9)	51 (56.0)	0.11	0.12
Employed	107 (42.8)	67 (42.1)	40 (44.0)		

*Note: p*‐value resulted from the independent samples *t*‐test for continuous variables and chi‐square test for categorical variables. Quantitative variables were presented as mean ± SD, and qualitative variables as frequency (percentage). Mean ± SD. *N* (%), *p* ≤ 0.05 is significant. Bold font in Table 1 is used to highlight the column headers (first row) to improve clarity and distinguish headers from the data.

Abbreviations: ALT, alanine aminotransferase; AST, aspartate aminotransferase; BFM, body fat mass; BMI, body mass index; BMR, basal metabolic rate; DBP, diastolic blood pressure; FBS, fasting blood sugar; FFM, fat free mass; HDL, high density lipoprotein; HOMA index, homeostatic model assessment for insulin resistance; hs‐CRP, high‐sensitivity C‐reactive protein; LDL, low density lipoprotein; SBP, systolic blood pressure; SLM, soft lean mass; SMM, skeletal muscle mass; TC, total cholesterol; TG, triglyceride; WC, waist circumference; WHR, waist hip ratio.

* *p*‐value was adjusted for age, BMI, total energy intake, and IPAQ by ANCOVA.

With adjustments for age, BMI, total energy intake, and physical activity, most differences remained significant. Specifically, the HTGW group continued to show elevated levels of body weight, BMR, WC, FFM, SMM, SLM, FBS, insulin, HOMA‐IR, TC, TG, LDL, diastolic blood pressure (DBP), and hs‐CRP (all *p* ≤ 0.05). Additionally, body fat mass (BFM; *p* = 0.004) and aspartate aminotransferase (AST; *p* = 0.001) were notably higher in the HTGW group post‐adjustment, while HDL cholesterol persisted at greater levels among the reference group (*p* = 0.009).

### 3.3 Dietary Intake Among the HTGW Phenotype

Comparisons of dietary intake, shown in Table 2, indicated significant differences in the consumption of specific nutrients. The HTGW group reported significantly higher intakes of iron (*p* = 0.04) and folate (*p* = 0.03), whereas the reference group had significantly higher vitamin E intake (*p* = 0.01). No meaningful differences were found for total energy intake or other macro‐ and micronutrients (*p* > 0.05).

**TABLE 2:** Dietary intake among the HTGW phenotype and the normal groups (*N* = 250).

	Normal *N* = 159	HTGW *N* = 91	*p*
Energy (kcal/day)	2640.32 ± 818.93	2584.11 ± 745.69	0.62
Protein (g/day)	87.91 ± 30.04	89.67 ± 26.88	0.69
Carbohydrate (g/day)	364.72 ± 116.50	379.56 ± 120.61	0.20
Fat (g/day)	94.31 ± 33.84	89.96 ± 31.06	0.17
Subtypes of fatty acids			
Cholesterol (g/day)	250.60 ± 108.22	257.20 ± 98.58	0.65
SFA (g/day)	27.82 ± 11.25	26.60 ± 10.39	0.26
MUFA (g/day)	31.69 ± 12.41	29.15 ± 10.30	0.09
PUFA (g/day)	20.30 ± 9.65	18.80 ± 7.48	0.26
Trans fatty acid (g/day)	0.001 ± 0.002	0.001 ± 0.001	0.88
Micronutrients			
Minerals			
Calcium (mg/day)	1152.75 ± 437.72	1142.15 ± 365.90	0.66
Iron (mg/day)	18.26 ± 5.96	19.39 ± 6.02	**0.04**
Magnesium (mg/day)	449.98 ± 145.06	482.84 ± 160.00	0.05
Zinc (mg/day)	12.65 ± 4.19	13.36 ± 4.40	0.09
Selenium (mg/day)	117.25 ± 42.14	126.50 ± 45.88	0.12
Phosphor (mg/day)	1617.01 ± 539.78	1648.80 ± 468.79	0.77
Vitamins			
C (mg/day)	185.13 ± 105.51	201.40 ± 117.48	0.32
D (μg/day)	2.06 ± 1.75	1.57 ± 1.19	0.05
E (mg/day)	18.01 ± 9.85	14.59 ± 5.59	**0.01**
B1 (mg/day)	2.04 ± 0.65	2.17 ± 0.68	0.07
B2 (mg/day)	2.15 ± 0.80	2.22 ± 0.74	0.49
B3 (mg/day)	25.10 ± 9.68	25.91 ± 8.58	0.57
B6 (mg/day)	2.14 ± 0.72	2.21 ± 0.73	0.55
B9 (mg/day)	591.89 ± 174.81	634.70 ± 197.36	**0.03**
B12 (mg/day)	4.32 ± 2.49	4.57 ± 2.55	0.41
Other nutrients			
Fiber (g/day)	44.20 ± 18.91	47.88 ± 19.26	0.18
Sugar (g/day)	139.35 ± 56.55	141.58 ± 58.17	0.99
Glucose (g/day)	20.66 ± 11.95	20.02 ± 10.20	0.51
Galactose (g/day)	2.78 ± 1.96	2.49 ± 1.83	0.35
Fructose (g/day)	24.92 ± 13.83	24.28 ± 12.53	0.54
Sucrose (g/day)	31.83 ± 18.62	33.45 ± 23.65	0.61

*Note:* Variables were presented as mean ± SD. Nutrients were adjusted for age, BMI, total energy intake, and IPAQ. *p* ≤ 0.05 is significant. Bold font in Table 2 is used to highlight the column headers (first row) to improve clarity and distinguish headers from the data.

Abbreviations: DFE, dietary folate equivalents; HTGW, hypertriglyceridemic waist phenotype; MUFA, monounsaturated fatty Acid; PUFA, polyunsaturated fatty acid; SFA, saturated fatty acid.

### 3.4 Food Insecurity and the Odds of the HTGW Phenotype

As presented in Table 3, experiencing food insecurity was strongly associated with higher odds of exhibiting the HTGW phenotype. In the unadjusted model, women with food insecurity demonstrated over twice the odds of presenting with the HTGW phenotype compared to their food‐secure counterparts (OR: 2.24; 95% CI: 1.19–4.20; *p* = 0.01). This association persisted after adjusting for age, BMI, total energy intake, and physical activity, with food‐insecure women exhibiting significantly higher odds of the HTGW phenotype (OR: 2.13; 95% CI: 1.06–4.28; *p* = 0.03).

**TABLE 3:** The association between food insecurity and HTGW phenotype (*N* = 250).

Models	OR	95% Cl	*p*
Crude model			
Normal	Reference group		
HTGW phenotype	2.24	1.19–4.20	**0.01**
Adjusted model			
Normal	Reference group		
HTGW phenotype	2.13	1.06–4.28	**0.03**

*Note:* Bold font in Table 3 is used to highlight the column headers (first row) to improve clarity and distinguish headers from the data.

## 4 Discussion

This cross‐sectional investigation explored whether household food insecurity is linked to the hypertriglyceridemic waist (HTGW) phenotype among Iranian women with overweight or obesity. Women reporting food insecurity were over twice as likely to present with HTGW relative to women in food‐secure households. Previous research has not explicitly focused on this link within overweight and obese Iranian women, making the present study novel in this regard.

Multiple studies have reported a persistent link between food insecurity and detrimental health effects, including impaired weight regulation and elevated risks of diabetes, hypertension, dyslipidemia, coronary heart disease, stroke, and cardiovascular mortality (Li and Rosenthal 2021). The HTGW phenotype, meanwhile, serves as a practical clinical marker for a cluster of metabolic disturbances such as hyperinsulinemia, elevated apolipoprotein B (Apo B), and high LDL cholesterol frequently associated with insulin resistance (Esmaillzadeh and Azadbakht 2010). Prior evidence suggests that nearly one‐third of Iranian women (32%) present with this phenotype (Amini et al. 2011). Our findings correspond with studies showing that food insecurity is linked to central adiposity and lipid abnormalities. For instance, a cross‐sectional analysis reported that adolescents from food‐insecure households had significantly higher waist circumference (WC) *z*‐scores compared with their food‐secure peers (Poulsen et al. 2019). In another study, Myers et al. (2019) demonstrated that food‐insecure women exhibited higher BMI and WC, while no significant association was observed among men. This gender disparity may reflect women's greater propensity to consume energy‐dense foods in response to food insecurity–related stress (Li and Rosenthal 2021; Habhab et al. 2009). In addition, women may be more inclined to adopt unhealthy eating patterns when they perceive household food shortages (Li and Rosenthal 2021). Evidence from NHANES (2007–2014) further indicated that women with low food security were significantly more likely to have a WC ≥ 88 cm and TG ≥ 150 mg/dL (Park and Strauss 2020). Consistent findings were also reported by Maldonado et al. (2022), who showed that Hispanic/Latino children experiencing food insecurity had higher TG levels, while U.S. data revealed that food‐insecure women without hunger were more likely to exhibit abnormal TG concentrations (Tayie and Zizza 2009).

Nevertheless, the literature remains inconclusive. Several studies have reported no significant association between food insecurity and indicators of metabolic risk. A recent meta‐analysis reported no significant association between food insecurity and dyslipidemia in adults (Arenas et al. 2022). Similarly, Theodoridis et al. (2018) found no relationship between levels of food insecurity and WC among university students, and comparable inconsistencies were noted in Inuit populations (Huet et al. 2012). Likewise, Faramarzi et al. (2019) did not detect significant links between food insecurity, WC, and TG. In contrast, Shariff et al. (2014) suggested an inverse association, observing a lower prevalence of abdominal obesity or TG abnormalities in food‐insecure groups.

Several explanations may account for these heterogeneous findings. Variations in food insecurity measurement tools, study designs, participant characteristics, and physical activity levels are likely contributors (Jafari et al. 2017). Second, mental health plays a mediating role: food insecurity often coexists with psychological distress, which in turn can drive unhealthy dietary patterns (Bruening et al. 2016). Moreover, alterations in lipid profiles may require prolonged exposure to food insecurity, meaning short‐term studies could fail to capture these outcomes (Arenas et al. 2022).

**FIGURE 1:**
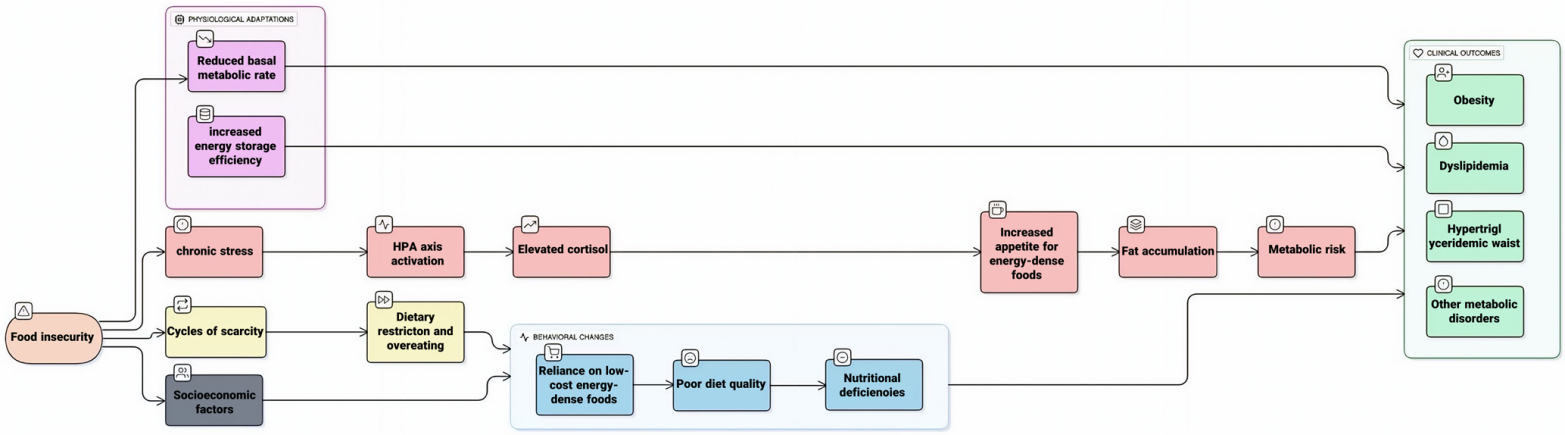
Conceptual model of mechanisms underlying the association between food insecurity and HTGW phenotype.

Multiple mechanisms may underlie the observed relationship between food insecurity and HTGW (Figure 1). Periods of scarcity alternating with relative adequacy may lead to cycles of restriction and overeating (Shariff et al. 2014). Food insecurity often leads individuals to rely on low‐cost, energy‐dense foods, resulting in poorer diet quality and a greater risk of chronic diseases (Castro et al. 2022; Angeles‐Agdeppa et al. 2021). Additionally, adaptive physiological responses to scarcity, such as reductions in basal metabolic rate, may enhance energy storage efficiency (Faramarzi et al. 2019). Chronic stress induced by food insecurity can activate the hypothalamic–pituitary–adrenal axis, leading to increased cortisol secretion, which in turn enhances appetite for energy‐dense foods, promotes fat accumulation, and elevates metabolic risk (Crowder et al. 2021; Bateson and Pepper 2023; Jaschke and Wang 2023). Together, these pathways contribute to the development of obesity, dyslipidemia, and other metabolic disorders.

Although earlier studies have reported links between food insecurity and cardiometabolic risk factors (Zierath et al. 2023), including central adiposity and lipid abnormalities, in diverse populations (Poulsen et al. 2019), this investigation is the first to specifically examine the relationship between food insecurity and the HTGW phenotype among overweight and obese Iranian women. It is further strengthened by an adequate sample size and adjustment for several potential confounders. However, certain constraints of the study should be noted. The cross‐sectional design precludes causal inference. In addition, the relatively small sample size restricted our analysis to a simplified two‐group classification of the HTGW phenotype and its reference group. Consistent with this approach, He et al. (52) also categorized participants into only two groups, HTGW and non‐HTGW, when examining the risk of future diabetes, thereby supporting the validity of such dichotomous grouping. Dietary intake was assessed using an FFQ, which is subject to recall bias. Measurement errors cannot be entirely ruled out, and residual confounding remains a possibility despite adjustments. Moreover, the study was limited to overweight and obese women in Tehran, which restricts the external validity of the findings. Specifically, our participants had a BMI between 25 and 40 kg/m^2^, so the results may not be directly applicable to normal‐weight individuals.

In addition, because all participants were women, the observed association between food insecurity and the HTGW phenotype might not apply to men. Previous studies have also reported gender differences in the food insecurity–obesity paradox, with stronger associations often seen in women. Moreover, despite adjusting our ANCOVA models for major confounders, including age, BMI, total energy intake, and physical activity, we did not apply statistical corrections for multiple comparisons across the specific nutrient intakes presented in Table 2. Consequently, the precise *p*‐values observed for certain nutrients, such as iron (*p* = 0.04) and folate (*p* = 0.03), should be interpreted cautiously.

Finally, as participants were recruited from health centers in Tehran, the findings may not represent populations from other geographic regions or countries with different cultural or socioeconomic contexts. Future studies with more diverse and representative samples are warranted to confirm these results.

## 5 Conclusion

Our findings reveal a meaningful relationship between food insecurity and the HTGW phenotype among overweight and obese Iranian women. Food insecurity emerges as an important social factor contributing to metabolic disturbances, adding new evidence to the discussion on the food insecurity–obesity paradox within non‐Western populations. These results emphasize the importance of implementing context‐specific nutritional, behavioral, and policy measures to confront the combined challenges of food insecurity and cardiometabolic disorders. At the same time, the cross‐sectional design restricts causal interpretation. Future prospective and interventional research is needed to substantiate these observations, clarify underlying biological and behavioral pathways, and support the development of evidence‐based strategies to mitigate the metabolic consequences of food insecurity in diverse settings.

## Author Contributions

P.J. and A.M. conceived and coordinated the project. P.J., A.D., N.V., and M.G. contributed to writing and experimental procedures. F.A. carried out statistical analyses. C.C.T.C. handled editing and revision of the manuscript. K.M. and A.M. supervised the study and reviewed and approved the final manuscript. All authors reviewed, revised, and approved the final manuscript.

## Funding

This work was supported by Tehran University of Medical Sciences and Health Services, 1402‐1‐125‐64675.

## Ethics Statement

Ethical approval was granted by the Ethics Committee of Tehran University of Medical Sciences (IR.TUMS.MEDICINE.REC.1402.172). We confirm that all procedures conducted in this study were carried out in accordance with applicable guidelines and regulations.

## Consent

All participants provided written informed consent before enrollment.

## Conflicts of Interest

The authors declare no conflicts of interest.

## Data Availability

The data supporting the findings of this study are available from Dr. Khadijeh Mirzaei; however, there are restrictions on their accessibility, as they were used under license for this specific study and are not publicly available. However, the data can be obtained from the authors upon reasonable request, provided that Dr. Khadijeh Mirzaei grants permission.

## References

[fsn371244-bib-0001] Abbade, E. B. , and H. J. F. S. Dewes . 2015. “Food Insecurity Worldwide Is Derived From Food Supply Patterns.” 7, no. 1: 109–120.

[fsn371244-bib-0002] Amini, M. , A. Esmaillzadeh , M. Sadeghi , N. Mehvarifar , M. Amini , and M. Zare . 2011. “The Association of Hypertriglyceridemic Waist Phenotype With Type 2 Diabetes Mellitus Among Individuals With a First‐Degree Relative History of Diabetes.” Journal of Research in Medical Sciences: The Official Journal of Isfahan University of Medical Sciences 16, no. 2: 156. 22091225 PMC3214297

[fsn371244-bib-0003] Angeles‐Agdeppa, I. , M. B. Toledo , and J. A. T. Zamora . 2021. “Moderate and Severe Level of Food Insecurity Is Associated With High Calorie‐Dense Food Consumption of Filipino Households.” Journal of Nutrition and Metabolism 2021, no. 1: 5513409. 34777860 10.1155/2021/5513409 PMC8580648

[fsn371244-bib-0004] Arenas, D. J. , S. Beltrán , M. Pharel , I. López‐Hinojosa , G. Vilá‐Arroyo , and H. M. DeLisser . 2022. “A Systematic Review and Meta‐Analysis of Food Insecurity and Dyslipidemia.” Journal of the American Board of Family Medicine 35, no. 4: 656–667. 35896471 10.3122/jabfm.2022.04.210413

[fsn371244-bib-0005] Barrett, C. B. J. S. 2010. “Measuring Food Insecurity.” Science 327, no. 5967: 825–828. 20150491 10.1126/science.1182768

[fsn371244-bib-0006] Bateson, M. , and G. V. Pepper . 2023. “Food Insecurity as a Cause of Adiposity: Evolutionary and Mechanistic Hypotheses.” Philosophical Transactions of the Royal Society, B: Biological Sciences 378, no. 1888: 20220228. 10.1098/rstb.2022.0228 PMC10475876 37661744

[fsn371244-bib-0007] Behzadifar, M. , M. Behzadifar , S. Abdi , et al. 2016. “Prevalence of Food Insecurity in Iran: A Systematic Review and Meta‐Analysis.” Archives of Iranian Medicine 19: 288–294. 27041526

[fsn371244-bib-0008] Bergler‐Klein, J. J. W. K. W. 2019. “What's New in the ESC 2018 Guidelines for Arterial Hypertension: The Ten Most Important Messages.” Wiener Klinische Wochenschrift 131, no. 7–8: 180–185. 30715608 10.1007/s00508-018-1435-8 PMC6459798

[fsn371244-bib-0009] Bickel, G. , M. Nord , C. Price , W. Hamilton , and J. Cook . 2000. “Guide to Measuring Household Food Security.”

[fsn371244-bib-0010] Bruening, M. , S. Brennhofer , I. Van Woerden , M. Todd , and M. Laska . 2016. “Factors Related to the High Rates of Food Insecurity Among Diverse, Urban College Freshmen.” Journal of the Academy of Nutrition and Dietetics 116, no. 9: 1450–1457. 27212147 10.1016/j.jand.2016.04.004 PMC5520984

[fsn371244-bib-0011] Castro, M. A. D. , M. D. M. Fontanelli , C. A. Nogueira‐de‐Almeida , and M. Fisberg . 2022. “Food Insecurity Reduces the Chance of Following a Nutrient‐Dense Dietary Pattern by Brazilian Adults: Insights From a Nationwide Cross‐Sectional Survey.” Nutrients 14, no. 10: 2126. 35631267 10.3390/nu14102126 PMC9143026

[fsn371244-bib-0012] Chang, R. , Z. Javed , M. Taha , et al. 2022. “Food Insecurity and Cardiovascular Disease: Current Trends and Future Directions.” American Journal of Preventive Cardiology 9: 100303. 34988538 10.1016/j.ajpc.2021.100303 PMC8702994

[fsn371244-bib-0013] Craig, C. L. , A. L. Marshall , M. Sjöström , et al. 2003. “International Physical Activity Questionnaire: 12‐Country Reliability and Validity.” 35, no. 8: 1381–1395. 10.1249/01.MSS.0000078924.61453.FB 12900694

[fsn371244-bib-0014] Crespo‐Bellido, M. S. , S. K. Grutzmacher , Y. Takata , and E. J. Smit . 2021. “The Association Between Food‐Away‐From‐Home Frequency and a Higher BMI Varies by Food Security Status in US Adults.” Journal of Nutrition 151, no. 2: 387–394. 33296463 10.1093/jn/nxaa364

[fsn371244-bib-0015] Crowder, S. L. , T. Beckie , and M. Stern . 2021. “A Review of Food Insecurity and Chronic Cardiovascular Disease: Implications During the COVID‐19 Pandemic.” Ecology of Food and Nutrition 60, no. 5: 596–611. 34617867 10.1080/03670244.2021.1956485 PMC9632247

[fsn371244-bib-0016] Dave, J. M. , A. E. Evans , R. P. Saunders , K. W. Watkins , and K. A. J. J. A. D. A. Pfeiffer . 2009. “Associations Among Food Insecurity, Acculturation, Demographic Factors, and Fruit and Vegetable Intake at Home in Hispanic Children.” 109, no. 4: 697–701. 10.1016/j.jada.2008.12.017 19328265

[fsn371244-bib-0017] Dietz, W. H. 1995. “Does Hunger Cause Obesity?” Pediatrics 95, no. 5: 766–767. 7724321

[fsn371244-bib-0018] El Zein, A. , S. E. Colby , W. Zhou , et al. 2020. “Food Insecurity Is Associated With Increased Risk of Obesity in US College Students.” Current Developments in Nutrition 4, no. 8: nzaa120. 32793850 10.1093/cdn/nzaa120 PMC7408225

[fsn371244-bib-0019] Esmaillzadeh, A. , and L. Azadbakht . 2010. “Increased Levels of Inflammation Among Women With Enlarged Waist and Elevated Triglyceride Concentrations.” Annals of Nutrition and Metabolism 57, no. 2: 77–84. 20733290 10.1159/000318588

[fsn371244-bib-0020] Faramarzi, E. , M. Somi , A. Ostadrahimi , et al. 2019. “Association Between Food Insecurity and Metabolic Syndrome in the North West of Iran: Azar Cohort Study.” Journal of Cardiovascular and Thoracic Research 11, no. 3: 196. 31579459 10.15171/jcvtr.2019.33 PMC6759622

[fsn371244-bib-0021] Fernández‐García, J. C. , A. Muñoz‐Garach , M. Á. Martínez‐González , et al. 2020. “Association Between Lifestyle and Hypertriglyceridemic Waist Phenotype in the PREDIMED‐Plus Study.” Obesity 28, no. 3: 537–543. 32090511 10.1002/oby.22728

[fsn371244-bib-0022] Habhab, S. , J. P. Sheldon , and R. C. Loeb . 2009. “The Relationship Between Stress, Dietary Restraint, and Food Preferences in Women.” Appetite 52, no. 2: 437–444. 19135112 10.1016/j.appet.2008.12.006

[fsn371244-bib-0023] Heshmati, J. , F. Golab , M. Morvaridzadeh , et al. 2020. “The Effects of Curcumin Supplementation on Oxidative Stress, Sirtuin‐1, and Peroxisome Proliferator‐Activated Receptor γ Coactivator 1α Gene Expression in Polycystic Ovarian Syndrome (PCOS) Patients: A Randomized Placebo‐Controlled Clinical Trial.” Diabetes and Metabolic Syndrome: Clinical Research and Reviews 14, no. 2: 77–82. 10.1016/j.dsx.2020.01.002 31991296

[fsn371244-bib-0024] Huet, C. , R. Rosol , and G. M. Egeland . 2012. “The Prevalence of Food Insecurity Is High, and the Diet Quality Is Poor in Inuit Communities.” Journal of Nutrition 142, no. 3: 541–547. 22323760 10.3945/jn.111.149278

[fsn371244-bib-0025] IFAD UN . 2017. The State of Food Security and Nutrition in the World. FAO.

[fsn371244-bib-0026] Ivers, L. C. , and K. A. Cullen . 2011. “Food Insecurity: Special Considerations for Women.” American Journal of Clinical Nutrition 94, no. 6: 1740S–1744S. 22089447 10.3945/ajcn.111.012617 PMC3226027

[fsn371244-bib-0027] Jafari, F. , S. Ehsani , A. Nadjarzadeh , A. Esmaillzadeh , M. Noori‐Shadkam , and A. Salehi‐Abargouei . 2017. “Household Food Insecurity Is Associated With Abdominal but Not General Obesity Among Iranian Children.” BMC Public Health 17, no. 1: 1–8. 28431549 10.1186/s12889-017-4262-3 PMC5399801

[fsn371244-bib-0028] Janghorbani, M. , M. R. Salamat , A. Aminorroaya , and M. Amini . 2017. “Utility of the Visceral Adiposity Index and Hypertriglyceridemic Waist Phenotype for Predicting Incident Hypertension.” Endocrinology and Metabolism 32, no. 2: 221–229. 28537054 10.3803/EnM.2017.32.2.221 PMC5503867

[fsn371244-bib-0029] Jaschke, N. P. , and A. Wang . 2023. “The Neurocircuitry of Fasting‐Induced Glucocorticoid Release.” Cell Metabolism 35, no. 9: 1497–1499. 37673035 10.1016/j.cmet.2023.08.004

[fsn371244-bib-0030] Li, Y. , and S. R. Rosenthal . 2021. “Food Insecurity and Obesity Among US Young Adults: The Moderating Role of Biological Sex and the Mediating Role of Diet Healthfulness.” Public Health Nutrition 24, no. 15: 5058–5065. 33183390 10.1017/S1368980020004577 PMC11082795

[fsn371244-bib-0031] Maldonado, L. E. , D. Sotres‐Alvarez , J. Mattei , et al. 2022. “Food Insecurity and Cardiometabolic Markers: Results From the Study of Latino Youth.” Pediatrics 149, no. 4: e2021053781. 35292821 10.1542/peds.2021-053781 PMC9595113

[fsn371244-bib-0032] Moghaddam, M. B. , F. B. Aghdam , M. A. Jafarabadi , H. Allahverdipour , S. D. Nikookheslat , and S. Safarpour . 2012. “The Iranian Version of International Physical Activity Questionnaire (IPAQ) in Iran: Content and Construct Validity, Factor Structure, Internal Consistency and Stability.” World Applied Sciences Journal 18, no. 8: 1073–1080.

[fsn371244-bib-0033] Mohajeri, M. , and R. J. M. J. N. Mohajery . 2022. “Food Security is Associated With Some Risk Factors of Obesity‐Related Diseases in the Ardabil‐Iran Population.” Mediterranean Journal of Nutrition and Metabolism 15: 1–9.

[fsn371244-bib-0034] Mohammadi, F. , N. Omidvar , G. G. Harrison , et al. 2013. “Is Household Food Insecurity Associated With Overweight/Obesity in Women?” Iranian Journal of Public Health 42, no. 4: 380–390. 23785677 PMC3684724

[fsn371244-bib-0035] Mohammadi, F. , N. Omidvar , A. Houshiar‐Rad , M.‐R. Khoshfetrat , M. Abdollahi , and Y. Mehrabi . 2012. “Validity of an Adapted Household Food Insecurity Access Scale in Urban Households in Iran.” Public Health Nutrition 15, no. 1: 149–157. 21806860 10.1017/S1368980011001376

[fsn371244-bib-0036] Myers, C. A. , C. K. Martin , R. L. Newton Jr. , et al. 2019. “Cardiovascular Health, Adiposity, and Food Insecurity in an Underserved Population.” Nutrients 11, no. 6: 1376. 31248113 10.3390/nu11061376 PMC6628173

[fsn371244-bib-0037] Parikh, R. M. , and V. Mohan . 2012. “Changing Definitions of Metabolic Syndrome.” Indian Journal of Endocrinology and Metabolism 16, no. 1: 7. 22276247 10.4103/2230-8210.91175 PMC3263200

[fsn371244-bib-0038] Park, S. H. , and S. M. Strauss . 2020. “Food Insecurity as a Predictor of Metabolic Syndrome in US Female Adults.” Public Health Nursing 37, no. 5: 663–670. 32729129 10.1111/phn.12781

[fsn371244-bib-0039] Parker, E. D. , R. Widome , J. A. Nettleton , and M. A. J. A. Pereira . 2010. “Food Security and Metabolic Syndrome in US Adults and Adolescents: Findings From the National Health and Nutrition Examination Survey, 1999–2006.” Annals of Epidemiology 20, no. 5: 364–370. 20382337 10.1016/j.annepidem.2010.02.009 PMC5842796

[fsn371244-bib-0040] Poulsen, M. N. , L. Bailey‐Davis , J. Pollak , A. G. Hirsch , and B. S. Schwartz . 2019. “Household Food Insecurity and Home Food Availability in Relation to Youth Diet, Body Mass Index, and Adiposity.” Journal of the Academy of Nutrition and Dietetics 119, no. 10: 1666–1675. 30858071 10.1016/j.jand.2019.01.001 PMC6732246

[fsn371244-bib-0041] Rasmusson, G. , J. A. Lydecker , J. A. Coffino , M. A. White , and C. M. Grilo . 2019. “Household Food Insecurity Is Associated With Binge‐Eating Disorder and Obesity.” International Journal of Eating Disorders 52, no. 1: 28–35. 10.1002/eat.22990 PMC6584603 30565270

[fsn371244-bib-0042] Salinas‐Roca, B. , L. Rubió‐Piqué , E. Carrillo‐Álvarez , and G. Franco‐Alcaine . 2022. “Impact of Health and Social Factors on the Cardiometabolic Risk in People With Food Insecurity: A Systematic Review.” International Journal of Environmental Research and Public Health 19, no. 21: 14447. 36361326 10.3390/ijerph192114447 PMC9655931

[fsn371244-bib-0043] Savari, M. , L. Barfizadeh , and Z. Asadi . 2021. “Effects of Social Capital on Achieving Food Security in Drought Conditions (Case Study: Rural Settlements in Dorud County).” Geography and Environmental Planning 32, no. 4: 1–28.

[fsn371244-bib-0044] Shariff, Z. M. , N. Sulaiman , R. A. Jalil , et al. 2014. “Food Insecurity and the Metabolic Syndrome Among Women From Low‐Income Communities in Malaysia.” Asia Pacific Journal of Clinical Nutrition 23, no. 1: 138–147. 24561982 10.6133/apjcn.2014.23.1.05

[fsn371244-bib-0045] Tabibian, S. , E. Daneshzad , N. Bellissimo , et al. 2018. “Association Between Adherence to the Dietary Approaches to Stop Hypertension Diet With Food Security and Weight Status in Adult Women.” Nutrition & Dietetics 75, no. 5: 481–487. 29888435 10.1111/1747-0080.12440

[fsn371244-bib-0046] Tayie, F. A. , and C. A. Zizza . 2009. “Food Insecurity and Dyslipidemia Among Adults in the United States.” Preventive Medicine 48, no. 5: 480–485. 19285104 10.1016/j.ypmed.2009.03.003

[fsn371244-bib-0047] Te Vazquez, J. , S. N. Feng , C. J. Orr , and S. A. Berkowitz . 2021. “Food Insecurity and Cardiometabolic Conditions: A Review of Recent Research.” Current Nutrition Reports 10, no. 4: 243–254. 34152581 10.1007/s13668-021-00364-2 PMC8216092

[fsn371244-bib-0048] Theodoridis, X. , M. Grammatikopoulou , K. Gkiouras , et al. 2018. “Food Insecurity and Mediterranean Diet Adherence Among Greek University Students.” Nutrition, Metabolism, and Cardiovascular Diseases 28, no. 5: 477–485. 10.1016/j.numecd.2018.02.007 29655531

[fsn371244-bib-0049] Wang, H. H. , D. K. Lee , M. Liu , P. Portincasa , and D. Q. Wang . 2020. “Novel Insights Into the Pathogenesis and Management of the Metabolic Syndrome.” In Novel Insights Into the Pathogenesis and Management of the Metabolic Syndrome, vol. 23, 189–230. The Korean Society of Pediatric Gastroenterology, Hepatology and Nutrition. 10.5223/pghn.2020.23.3.189 PMC7231748 32483543

[fsn371244-bib-0050] Zheng, X. , X. Ren , M. Jiang , and L. Han . 2022. “Association Between Hypertriglyceridemic‐Waist Phenotype and Cardiovascular Disease: A Cohort Study and Meta‐Analysis.” Frontiers in Cardiovascular Medicine 9: 940168. 35990944 10.3389/fcvm.2022.940168 PMC9386422

[fsn371244-bib-0051] Zierath, R. , B. Claggett , M. E. Hall , et al. 2023. “Measures of Food Inadequacy and Cardiovascular Disease Risk in Black Individuals in the US From the Jackson Heart Study.” JAMA Network Open 6, no. 1: e2252055. 36689225 10.1001/jamanetworkopen.2022.52055 PMC9871801

